# Metabolic signatures and potential biomarkers of sarcopenia in suburb-dwelling older Chinese: based on untargeted GC–MS and LC–MS

**DOI:** 10.1186/s13395-024-00337-3

**Published:** 2024-03-07

**Authors:** Peipei Han, Chunhua Yuan, Xiaoyu Chen, Yuanqing Hu, Xiaodan Hu, Zhangtao Xu, Qi Guo

**Affiliations:** 1 Department of Rehabilitation Medicine, Shanghai University of Medicine and Health Sciences Affiliated Zhoupu Hospital, Shanghai, China; 2 College of Rehabilitation Sciences, Pudong New Area, Shanghai University of Medicine and Health Sciences, 279 Zhouzhu Highway, Shanghai, 201318 China; 3 Jiangwan Hospital of Shanghai Hongkou District, Shanghai University of Medicine and Health Science Affiliated First Rehabilitation Hospital, Shanghai, China; 4 Comprehensive Surgical Rehabilitation Ward, Shanghai Health Rehabilitation Hospital, Shanghai, China

**Keywords:** Biomarkers, GC–MS, LC–MS, Metabolomics, Sarcopenia

## Abstract

**Background:**

Untargeted metabolomics can be used to expand our understanding of the pathogenesis of sarcopenia. However, the metabolic signatures of sarcopenia patients have not been thoroughly investigated. Herein, we explored metabolites associated with sarcopenia by untargeted gas chromatography (GC)/liquid chromatography (LC)–mass spectrometry (MS) and identified possible diagnostic markers.

**Methods:**

Forty-eight elderly subjects with sarcopenia were age and sex matched with 48 elderly subjects without sarcopenia. We first used untargeted GC/LC–MS to analyze the plasma of these participants and then combined it with a large number of multivariate statistical analyses to analyze the data. Finally, based on a multidimensional analysis of the metabolites, the most critical metabolites were considered to be biomarkers of sarcopenia.

**Results:**

According to variable importance in the project (VIP > 1) and the *p*-value of *t*-test (*p* < 0.05), a total of 55 metabolites by GC–MS and 85 metabolites by LC–MS were identified between sarcopenia subjects and normal controls, and these were mostly lipids and lipid-like molecules. Among the top 20 metabolites, seven phosphatidylcholines, seven lysophosphatidylcholines (LysoPCs), phosphatidylinositol, sphingomyelin, palmitamide, L-2-amino-3-oxobutanoic acid, and palmitic acid were downregulated in the sarcopenia group; only ethylamine was upregulated. Among that, three metabolites of LysoPC(17:0), L-2-amino-3-oxobutanoic acid, and palmitic acid showed very good prediction capacity with AUCs of 0.887 (95% CI = 0.817–0.957), 0.836 (95% CI = 0.751–0.921), and 0.805 (95% CI = 0.717–0.893), respectively.

**Conclusions:**

These findings show that metabonomic analysis has great potential to be applied to sarcopenia. The identified metabolites could be potential biomarkers and could be used to study sarcopenia pathomechanisms.

## Introduction

Sarcopenia is a disease that is characterized by a decline in skeletal muscle mass, muscle strength, and physical performance [1]. It is associated with the aging process and is known to be associated with adverse health outcomes, such as disability, diabetes, metabolic syndrome, poor quality of life, and mortality [2]. Sarcopenia contributes to current increased health care costs and is becoming a major public health problem [3]. Accumulated evidence indicates that sarcopenia is multifactorial; some possible causes, such as neurological factors associated with loss of motor neurons, loss of muscle motor units, endocrine changes, and lifestyle changes associated with sedentary behavior and poor nutrition, might contribute to its onset and progression [4]. However, the biological mechanisms underlying the development of sarcopenia are still largely unknown. Therefore, novel approaches are needed to advance the understanding of the mechanisms of sarcopenia development.

Metabolomics is an emerging approach for identifying biomarkers to unveil the molecular mechanisms of complex diseases, for monitoring diseases, and for risk evaluation [5, 6]. Because metabolites represent the downstream expression of the genome, transcriptome, and proteome, their study is hence most powerful to reveal inherent omics variation closest to the disease risk/phenotype [7]. However, most of the current metabolomics studies have used a targeted approach, directed at specific candidate metabolites [8–10]. This approach restricts the potential to discover novel biomarkers and hitherto unknown pathways in sarcopenia development. Untargeted metabolomics has also been performed, such as untargeted profiling to identify trait-specific or shared metabolites associated with muscle mass and strength [11], to reveal specific metabolic profiles associated with decreased low skeletal muscle mass in postmenopausal women [12], and to compare the plasma metabolome [13]. However, they all used a single method; the recognition area of metabolites was relatively narrow. Therefore, the results of these studies had some limitations. Furthermore, studies on metabolomics in sarcopenia remain limited, especially in Asian populations. To better understand the pathogenesis of sarcopenia, we need to combine the two technologies and make full use of the technical advantages of gas chromatography–mass spectrometry (GC–MS) and liquid chromatography–mass spectrometry (LC–MS) to study the metabolomics of sarcopenia more comprehensively and accurately.

Here, we systematically investigated the relationships between plasma metabolites and sarcopenia using two untargeted metabolomics platforms: GC–MS and LC–MS. We aimed to understand the metabolic signature of sarcopenia, specifically for elderly individuals in Asian populations. We also attempted to identify potential sarcopenia associated metabolite biomarkers that differentiate elderly individuals with and without sarcopenia. The findings will help us better understand the development of sarcopenia and could assist in identifying new molecular targets for the treatment of the disease.

## Materials and methods

### Study participants

The research population included residents aged ≥ 65 years from Shanghai, China, who had joined China’s national free physical examination program. A total of 380 subjects had a plasma sample available at baseline. The study participants have been described in our previous study [14]. The design was a nested case–control study. Among the 380 subjects, 332 were normal older adults and 48 were patients with sarcopenia; we obtained 48 normal control (NC) subjects matched by age and sex using propensity score matching from the non-sarcopenia subjects. We employed nearest neighbor matching without replacement in a 1:1 manner. We used a caliper of 0.02 standard deviation of the logit of the propensity score. This study was approved by the Ethics Committee of Shanghai University of Medicine and Health Sciences. All participants voluntarily joined this study, provided written informed consent, and completed questionnaires that provided demographic information including age, sex, lifestyle factors, and medical history. Details of measurement methods have been described in our previous cross-sectional study [15].

### Assessment of sarcopenia

Sarcopenia was defined according to the Asian Working Group for Sarcopenia (AWGS) criteria [16], in which a person who has low muscle mass, low muscle strength, and/or low physical performance was identified as having sarcopenia. Low muscle mass was classified as relative skeletal muscle mass index (ASM/ht^2^) less than 7.0 kg/m^2^ and 5.7 kg/m^2^ in males and females, respectively; low muscle strength was defined as grip strength < 28 kg or < 18 kg for males and females, respectively; and low physical performance was defined as walking speed < 1.0 m/s for both males and females.

Muscle mass was measured using a direct segmental multi-frequency bioelectrical impedance analysis (BIA) (In-Body720; Biospace Co., Ltd., Seoul, Korea). Muscle strength was assessed by grip strength, measured using a dynamometer (GRIP-D; Takei Ltd, Niigata, Japan). Usual walking speed (m/s) on a 4-m course was used as an objective measure of physical performance. Details of measurement methods have been described in our previous cross-sectional study [15].

### Sample collection and processing

Each plasma sample was collected from the study subjects on an empty stomach in the morning and was then separated and stored in freezers at − 80 °C until the metabolomics assay. We thawed the samples at room temperature. First, 150 μL of plasma was added to a new Eppendorf tube, and 10 μL of L-2-chlorophenylalanine (0.3 mg/ml) with methanol dissolved in the tube was used as the internal standard. Next, a 450-μL mixture of methanol/acetonitrile (2/1) was added and vortexed for 1 min. The whole samples were extracted by ultrasonication for 10 min and stored at − 20 °C for 30 min. The extract was centrifuged for 10 min (4 °C, 13,000 RPM). A total of 200 μL of supernatant was dried in a freeze concentration centrifugal dryer, resolubilized by 300 μL of methanol/water (1/4), vortexed for 30 s, and extracted by ultrasonication for 3 min. After vigorous mixing, samples were centrifuged at 4 °C (13,000 rpm) for 10 min, and 150 μL of supernatants were filtered through 0.22-μm microfilters and transferred to LC vials. The vials were left at − 80 °C and then analyzed by LC–MS.

A total of 150 μL of sample was added to a 1.5-mL Eppendorf tube with 20 μL of 2 chloro-l-phenylalanine (0.3 mg/mL) dissolved in methanol as an internal standard, and the tube was vortexed for 10 s. Subsequently, 450 μL of an ice-cold mixture of methanol and acetonitrile (2/1, v/v) was added, and the mixtures were vortexed for 30 s, ultrasonicated in an ice water bath for 10 min, and stored at − 20 °C for 30 min. The extract was centrifuged at 13,000 rpm and 4 °C for 10 min. In a freeze concentration centrifugal dryer, 200 μL of supernatant was dried in a glass bottle. Then, 80 μL of 15 mg/mL methoxylamine hydrochloride in pyridine was subsequently added. The resultant mixture was vortexed vigorously for 2 min and incubated at 37 °C for 90 min. Then, 50 μL of BSTFA (with 1% TMCS) and 20 μL of n-hexane were added into the mixture, which was vortexed vigorously for 2 min and derivatized at 70 °C for 60 min. The samples were placed at ambient temperature for 30 min before GC–MS analysis.

### Metabolic profiling

The plasma sample preparation along with LC–MS analysis have been described in detail in our previous study [14]. Briefly, LC–MS analysis was performed on a liquid mass spectrometer system consisting of an ACQUITY ultra-performance liquid chromatography (UPLC) I-Class tandem VION IMS QT high-resolution mass spectrometer (Waters Corporation, Milford, USA). The samples were separated on the ACQUITY UPLC BEH C18 column (Waters Corporation; 1.7 μm, 100 × 2.1 mm) at a flow rate of 0.4 ml/min. The column was maintained at 45 °C, the sample chamber was set at 4 °C, and the injection volume was set to 1 μL. The mobile phases were water containing 0.1% formic acid (solution A) and acetonitrile/methanol (2/3, vol/vol) containing 0.1% formic acid (solution B). The gradient was 0–1 min, 30% B; 1–2.5 min, 30–60% B; 2.5–6.5 min, 60–90% B; 6.5–8.5 min, 90–100% B; 8.5–10.7 min, 100% B; 10.7–10.8 min, 100–1% B, 10.8–13 min, 1%B. The ion source was electrospray ionization (ESI), and the sample mass spectrometry signal acquisition was performed in positive and negative ion scanning mode, respectively. Mass spectrometric tuning parameters for LC–MS analysis employed optimized settings as follows: ion source temperature, 150 °C; capillary voltages, 2.5 kV; desolvation gas flow, 900 L/h; declustering potential, 40 V; collision energy, 4 eV; mass scan range, m/z 50–1,000; and scan time, 0.2 s [14].

A DB-5MSf used-silica capillary column (30 m × 0.25 mm × 0.25 μm, Agilent J& W Scientific, Folsom, CA, USA) was utilized to separate the derivatives; the derived samples were analyzed by GC–MS on an Agilent 7890B gas chromatography system coupled to an Agilent 5977A MSD system (Agilent Technologies Inc., CA, USA) [17]. In splitless mode, the injector temperature was held at 260 °C, and the injection volume was set at 1 μL. The initial oven temperature commenced at 60 °C held at 60 °C for 0.5 min and increased to 125 °C at a rate of 8 °C/min, followed by a ramp to 210 °C at a rate of 8 °C/min, further ramping to 270 °C at a rate of 15 °C/min, and ultimately reaching 305 °C at a rate of 20 C/min, where it was held for 5 min. The MS quadrupole and ion source (electron impact) were set at temperatures of 150 and 230 °C, respectively. Applying a collision energy of 70 eV, mass spectrometric data acquisition took place in full-scan mode (m/z 50–500) with a 5-min solvent delay time. Throughout the analytical run, quality control samples (QCs) were injected at regular intervals (every 10 samples) to generate a dataset for assessing repeatability.

### Data processing and analysis

The LC–MS data were processed by the software Progenesis QI version 2.3 (Nonlinear, Dynamics, Newcastle, UK) for meaningful data mining, performing peak alignment, picking, normalizing, and correcting the retention time (RT). The resulting matrix of features included information on the mass-to-charge ratio (m/z), RT, and peak intensities. The identification of compounds is based on the precise m/z, secondary fragments, and isotopic distribution, and the Human Metabolome Database (HMDB) (http://www.hmdb.ca/), LIPID MAPS (version 2.3) (http://www.lipidmaps.org/), Metabolite Mass Spectral Database (METLIN) (http://metlin.scripps.edu/), and self-built databases (EMDB) were used for qualitative analysis. The GC–MS data were imported into MS-DIAL software (version 2.74) for peak detection, peak identification, characterization, peak alignment, wave filtering, etc. The LUG database (Untargeted database of GC–MS rom Lumingbio) was used to characterize the metabolites. The three-dimensional matrix includes the following: sample information, the name of the peak of each substance, retention time, retention index, mass-to-charge ratio, and signal intensity. After screening, all peak signal intensities in each sample were segmented and normalized according to the internal standards with RSD > 0.3. Then, redundancy removal and peak merging were conducted to obtain the data matrix.

To understand the differences in metabolic profiles between the control and sarcopenia groups, principal component analysis (PCA) and orthogonal projection to latent structure with discriminant analysis (OPLS-DA) were used as a statistical analysis tool. To assess the OPLS-DA, two parameters, R2Y and Q2, are used. At the same time, the OPLS-DA model was cross validated by a 200-fold permutation test; the permutation test is evaluated by cross-validation, and the correlation coefficients R2 and Q2 of the cross-validation were used to verify whether there was overfitting [18].

Differential metabolites between groups were selected using a multidimensional couple with single-dimensional analysis. The variable importance in projection (VIP) generated in OPLS-DA represented differential metabolites with biological significance. Furthermore, the significance of differential metabolites was further verified by Student’s *t*-test. Variables with VIP > 1.0 and *p* < 0.05 were considered to be potential biomarkers of sarcopenia. The predictive performance of the model was assessed by estimating the area under the receiver operating characteristic (ROC) curve (AUC). At the same time, we also analyzed the correlation between the top 20 metabolites we screened and the components of sarcopenia (muscle mass, grip strength, and walking speed).

Baseline sociodemographic characteristics between the control and sarcopenia groups were compared using an independent *t*-test for numerical variables and the chi-squared test for categorical variables. Data with a normal distribution are expressed as the mean ± SD and categorical variables are expressed as proportions. Statistical analyses were performed using SPSS version 26.0 (SPSS Incorporation, Chicago, IL, USA) The significance standard was *p* < 0.05.

## Results

### Characteristics of the studied population

Among the 380 participants (161 men) who were available to be analyzed, 48 (12.6%) met the diagnostic criteria and were defined as having sarcopenia. The demographic and clinical characteristics of the study participants are presented in Table 1. The groups were matched for age and sex. There was no significant difference in the age distribution, sex, or the remaining indicators between the groups, which indicated that the subjects in each group were comparable.

**Table 1:** Baseline characteristics of studied groups

Characteristic	Sarcopenia group (*n* = 48)	Control group (*n* = 48)	*P* value
Age (years)	75.7 ± 5.8	74.1 ± 5.0	0.142
Sex (%)			
Male	39.6	39.6	1.000
Female	60.4	60.4	
ASM/Ht2 (kg/m^2^)	5.8 ± 0.8	7.0 ± 0.9	< 0.001
Grip strength (kg)	18.0 ± 6.1	26.8 ± 6.8	< 0.001
Walking test (m/s)	1.0 ± 0.3	1.2 ± 0.2	< 0.001
BMI (kg/m^2^)	19.9 ± 2.3	25.0 ± 2.7	< 0.005
MNA (score)	11.5 ± 1.3	13.3 ± 1.0	< 0.001
IPAQ (Met-min/week)	1209(3268–7031)	1883(4473–9698)	0.165
GDS (score)	6.7 ± 5.1	5.2 ± 3.6	0.093
Illiteracy (%)			0.575
No	81.3	87.5	
Yes	18.8	12.5	
Widowed (%)			0.150
No	68.8	83.3	
Yes	31.2	16.7	
Farming (%)			0.306
No	39.6	52.1	
Yes	60.4	47.9	
Smoking (%)			0.142
No	91.7	80.4	
Yes	8.3	19.6	
Drinking (%)			0.162
No	81.2	66.7	
Yes	18.8	33.3	
Diabetes (%)			
No	79.2	79.2	
Yes	20.8	20.8	
Hypertension (%)			1.000
No	29.2	29.2	
Yes	70.8	70.8	
Hyperlipidemia (%)			0.834
No	62.5	60.4	
Yes	37.5	39.6	
Stroke (%)			0.617
No	93.8	97.9	
Yes	6.3	2.1	
Heart disease (%)			0.883
No	67.4	66.0	
Yes	32.6	34.0	

Data are presented as mean ± SD for continuous variables and *n* (%) for categorical variables

### Metabolomics differences between studied groups

**Fig. 1:**
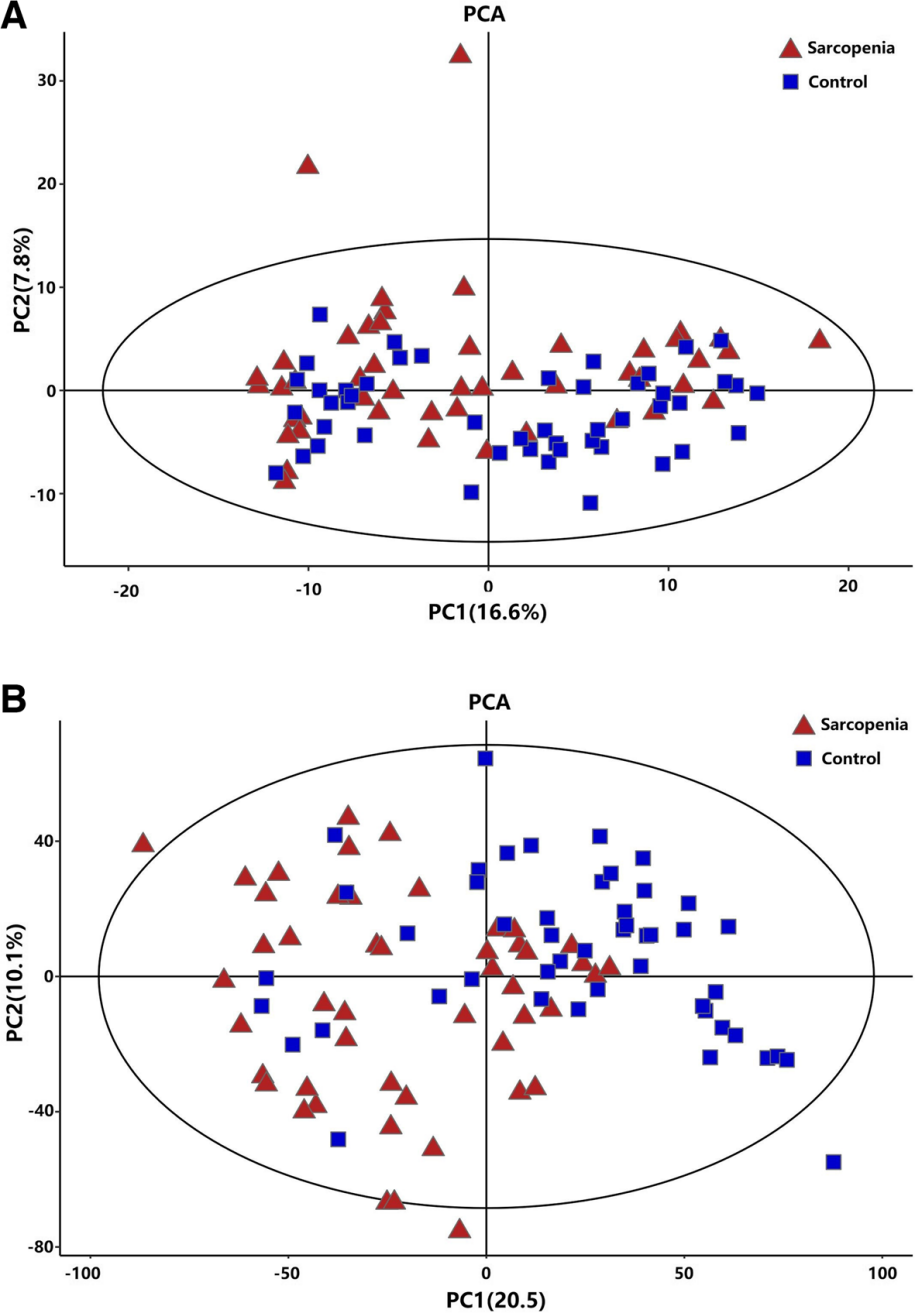
PCA analysis

**Fig. 2:**
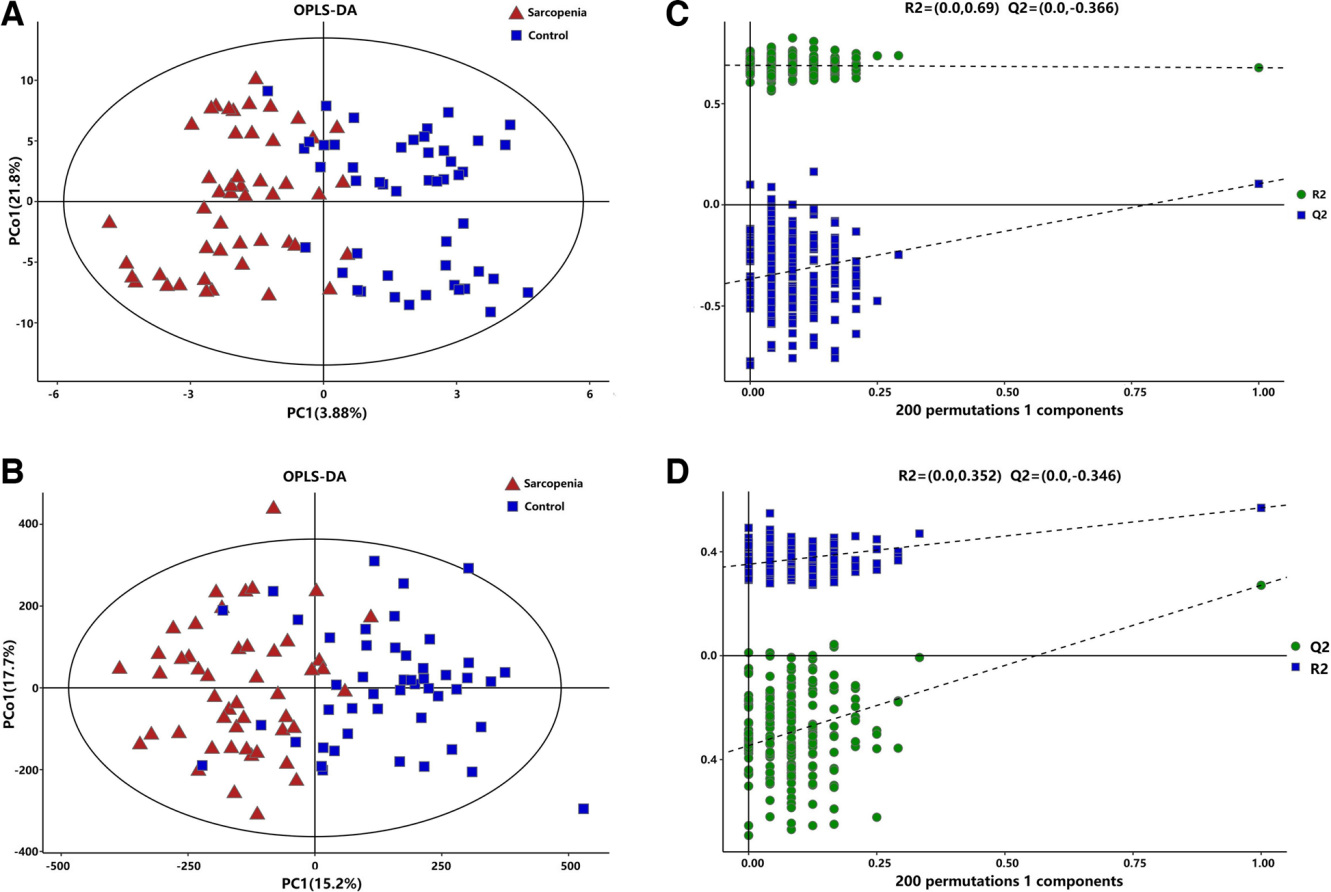
OPLS-DA on the metabolic spectrum

We performed a comprehensive metabolomics analysis of the plasma of the two groups. A total of 446 metabolites by GC–MS and 1009 metabolites by LC–MS were identified in plasma. The overall distribution trend of all samples was observed through PCA analysis (Fig. 1A, B). The total variance of the data represented by the first two principal components was 16.6% in GC–MS (Fig. 1A) and 20.6% in LC–MS (Fig. 1B). Considering the blank supervision of the PCA model, we constructed an OPLS-DA on the metabolic spectrum, and a tendency for separation was observed (Fig. 2A, B). The model was confirmed to not be overfitted following 200 permutation tests (Fig. 2C, D), which reveals that the model with good discrimination is predictive to be accurate and accurately defined.

**Fig. 3:**
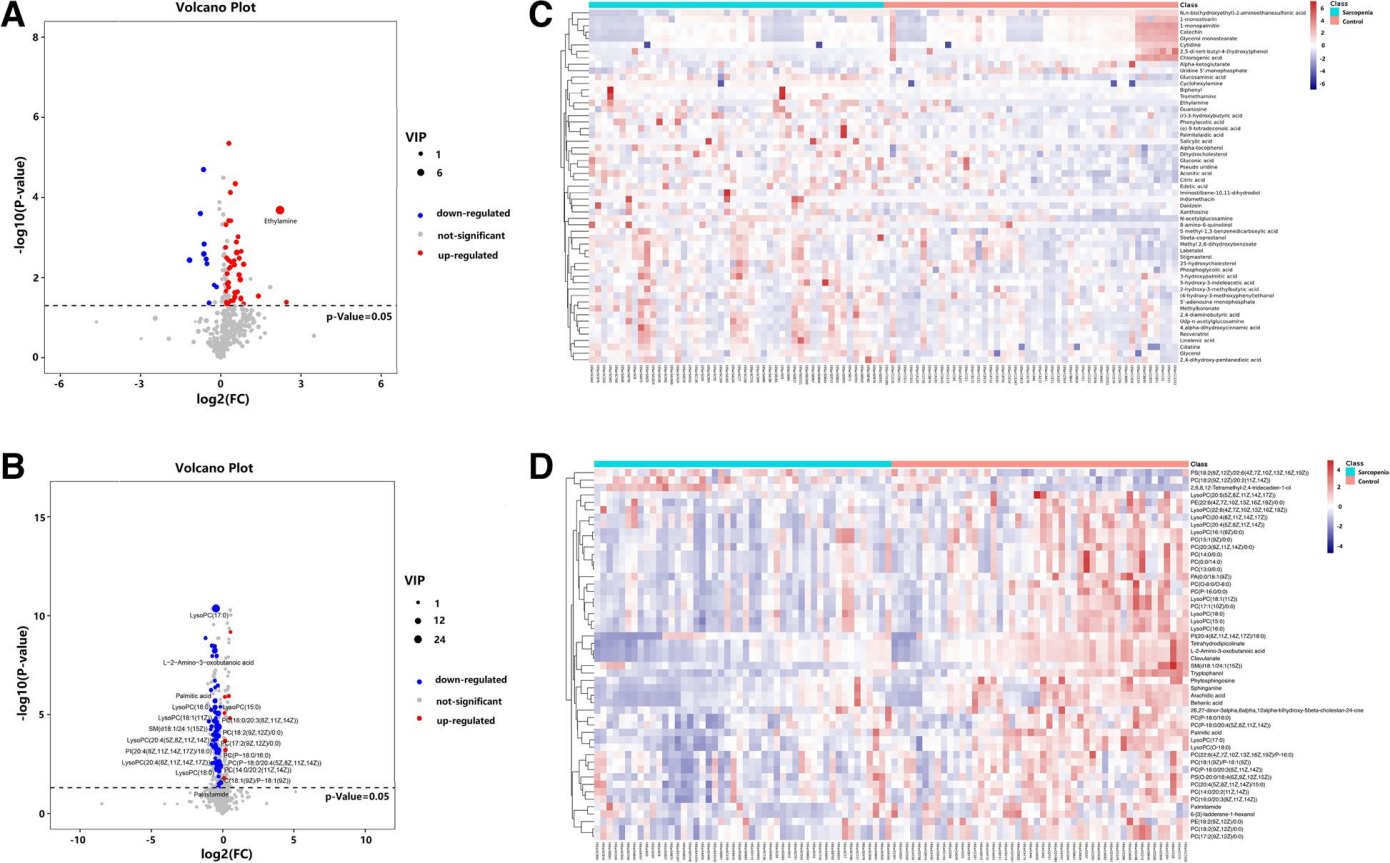
Volcano plot

**Fig. 4:**
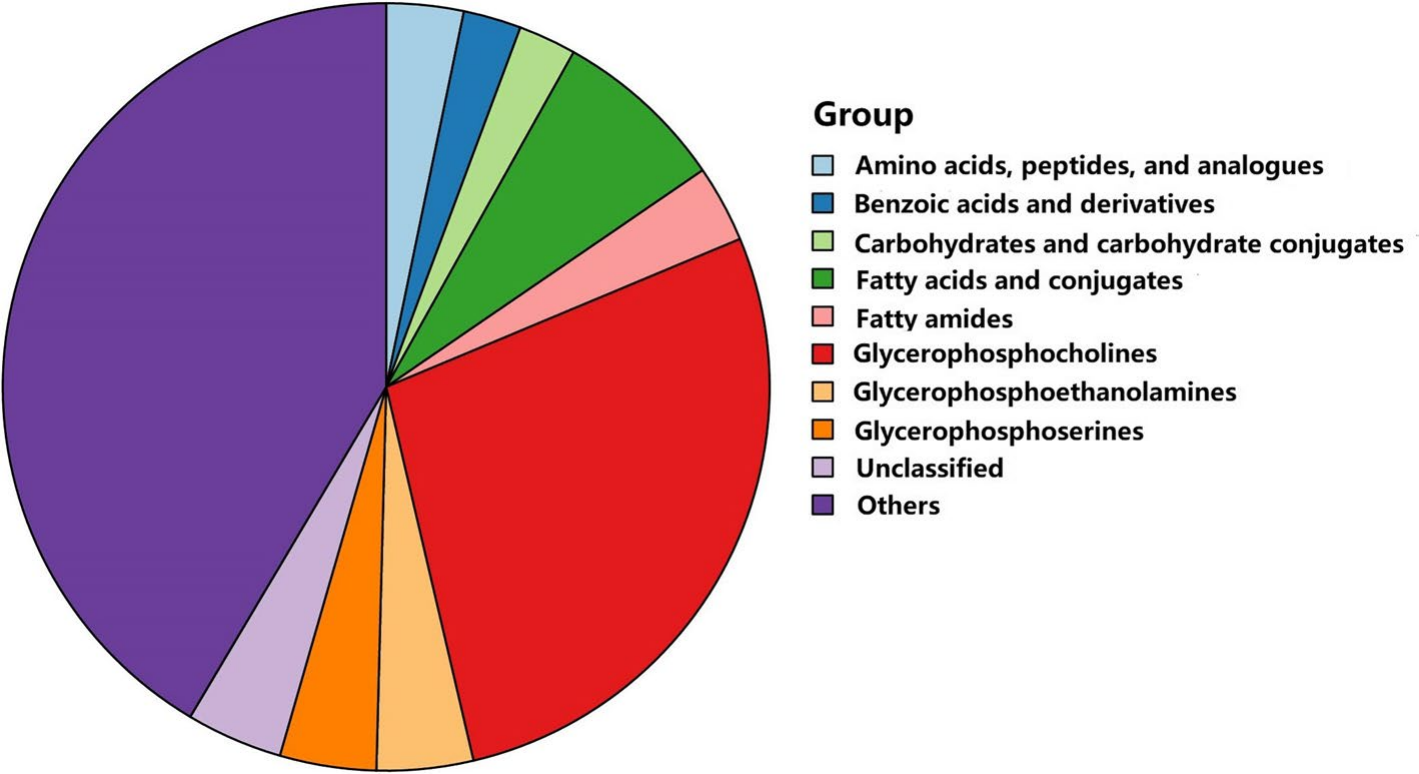
Classification of the metabolites

After multivariate analysis, 55 metabolites by GC–MS and 85 metabolites by LC–MS contributed significantly to the distinction of NC and sarcopenia with VIP values of > 1 and *p*-values of < 0.05. The volcano plot shows the *p* value and fold change value (Fig. 3A, B), thus proving the effectiveness of differential metabolites. The classification of the metabolites is shown in Fig. 4. Hierarchical clustering is carried out through the expression of all metabolites with significant differences (Fig. 3C, D), which can reflect the relationship among samples and the metabolite expression differences among different samples more directly. Figure 3 indicates that the differences in the metabolites we chose are significant. The VIP score identified the metabolites that contributed the most to the difference in metabolic profiles, and the top 20 metabolites designated by GC/LC–MS are shown in Table 2.

**Table 2:** The top 20 metabolites contributed most to the difference in metabolic profiles

Metabolite	Status^a^	VIP value^b^	FC^c^	Data origin	*p*^§^
PC(14:0/20:2(11Z,14Z))	↓	30.28	0.85	LC	0.003
LysoPC(17:0)	↓	24.63	0.73	LC	< 0.001
PC(16:0/20:3(8Z,11Z,14Z))	↓	20.39	0.81	LC	< 0.001
L-2-Amino-3-oxobutanoic acid	↓	10.24	0.69	LC	< 0.001
Ethylamine	**↑**	10.47	4.69	GC	< 0.001
LysoPC(15:0)	↓	8.60	0.81	LC	< 0.001
PC(18:2(9Z,12Z)/0:0)	↓	7.71	0.82	LC	< 0.001
LysoPC(18:1(11Z))	↓	6.46	0.75	LC	< 0.001
Palmitic acid	↓	5.78	0.70	LC	< 0.001
LysoPC(16:0)	↓	5.74	0.70	LC	< 0.001
PC(P-18:0/16:0)	↓	5.05	0.86	LC	0.002
PC(18:1(9Z)/P-18:1(9Z))	↓	4.86	0.83	LC	0.006
LysoPC(20:4(8Z,11Z,14Z,17Z))	↓	4.77	0.78	LC	0.003
PI(20:4(8Z,11Z,14Z,17Z)/18:0)	↓	4.77	0.74	LC	< 0.001
PC(17:2(9Z,12Z)/0:0)	↓	4.65	0.84	LC	< 0.001
SM(d18:1/24:1(15Z))	↓	4.60	0.73	LC	< 0.001
LysoPC(20:4(5Z,8Z,11Z,14Z))	↓	4.39	0.74	LC	< 0.001
PC(P-18:0/20:4(5Z,8Z,11Z,14Z))	↓	4.31	0.88	LC	0.004
LysoPC(18:0)	↓	4.19	0.82	LC	0.004
Palmitamide	↓	4.16	0.82	LC	0.048

^a^Relative concentrations compared to healthy controls: ↑ = upregulated, ↓ = downregulated

^b^Correlation coefficient and VIP value were obtained from OPLS-DA analysis

^c^Fold change between sarcopenia patients and healthy controls

^§^*P* value determined from Student’s *t*-test

### Evaluation of the metabolites panel for the diagnosis of sarcopenia

**Fig. 5:**
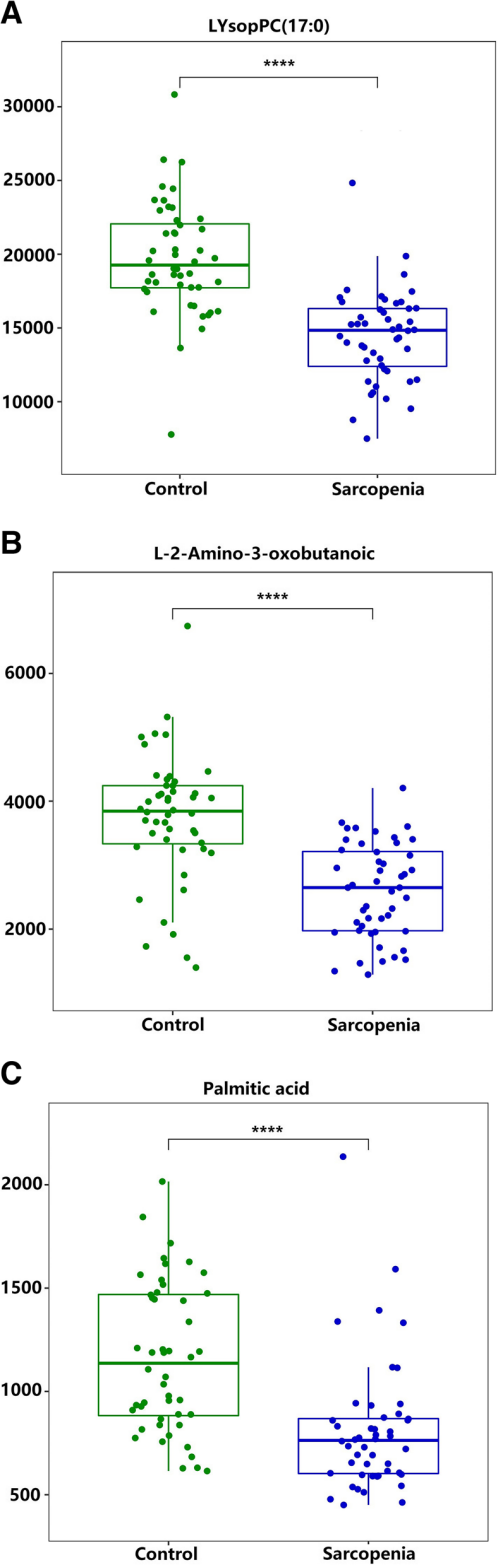
Top 3 potential biomarker box-and-whisker plots

Univariate ROC curve analysis was performed for the top 20 potential biomarkers. A total of 13 and 3 metabolites has areas under the ROC curve (AUC) of at least 0.7 and 0.8, respectively (Table 3). The top three metabolites that can discriminate the groups with the highest accuracy are LysoPC(17:0) (AUC = 0.887, 95% CI = 0.817–0.957), L-2-amino-3-oxobutanoic acid (AUC = 0.836, 95% CI = 0.751–0.921), and palmitic acid (AUC = 0.805, 95% CI = 0.717–0.893). Significant differences between the groups are also shown by the top 3 potential biomarker box-and-whisker plots (Fig. 5).

**Table 3:** The AUC values for metabolites

Metabolite	AUC	95% CI	Specificity	Sensitivity
LysoPC(17:0)	0.887	0.817–0.957	0.771	0.938
L-2-Amino-3-oxobutanoic acid	0.836	0.751–0.921	0.625	0.979
Palmitic acid	0.805	0.717–0.893	0.750	0.771
LysoPC(16:0)	0.761	0.665–0.857	0.792	0.646
LysoPC(15:0)	0.749	0.650–0.848	0.729	0.688
SM(d18:1/24:1(15Z))	0.748	0.649–0.847	0.729	0.688
LysoPC(18:1(11Z))	0.742	0.642–0.843	0.563	0.875
PC(16:0/20:3(8Z,11Z,14Z))	0.741	0.641–0.842	0.750	0.688
PC(18:2(9Z,12Z)/0:0)	0.721	0.619–0.824	0.604	0.771
Ethylamine	0.720	0.624–0.815	0.896	0.604
PI(20:4(8Z,11Z,14Z,17Z)/18:0)	0.717	0.612–0.822	0.688	0.771
LysoPC(20:4(5Z,8Z,11Z,14Z))	0.704	0.601–0.807	0.708	0.604
PC(17:2(9Z,12Z)/0:0)	0.701	0.596–0.806	0.583	0.771

### The correlations between components of sarcopenia and selected metabolites

**Fig. 6:**
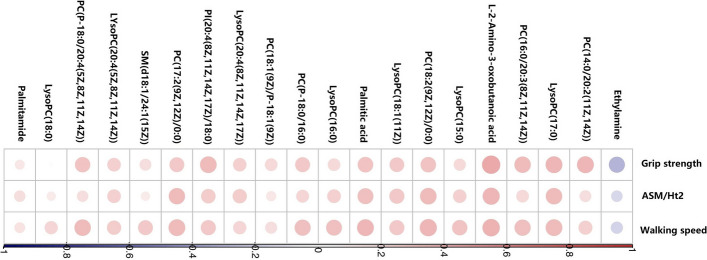
Correlations between the top 20 metabolites and components of sarcopenia

Figure 6 shows the correlations between the top 20 metabolites and components of sarcopenia (muscle mass, grip strength, and walking speed). Except for LysoPC(18:0), all of these metabolites were significantly correlated with the components of sarcopenia. Among the related metabolites, only ethylamine showed a significantly negative correlation. L-2-amino-3-oxobutanoic acid and LysoPC(17:0) were highly correlated with the three components of sarcopenia. The two metabolites are also the most discriminate.

## Discussion

Sarcopenia is a serious and major public health problem. However, the exact mechanism involved in sarcopenia is not well known. Metabolomics is widely regarded as the most phenotypic omics because it identifies and quantifies small molecular metabolites [19]. Because of its inherent sensitivity, metabolomics is the most powerful method to study local and specific stimulus responses and pathogeneses. Thus, in our study, untargeted GC–MS combined with LC–MS was used for the first time to determine the metabolomic signature of sarcopenia. Through multivariate analysis, we found that a large number of significant metabolites contributed to the distinction between NC and sarcopenia. In previous metabolomics studies, some potential biomarkers of sarcopenia have been found. These metabolites are mainly concentrated in fats [e.g., 12(S)-HETRE and 12(S)-HETE, arachidonic acid and glycerophosphocholine [11], linoleic acid, oleic acid, arachidonic acid, and 11, 14-eicosadienoic acid] and amino acids [e.g., aspartate, glutamic acid [11], N-acetyl-L-aspartic acid, carnosine [8], glutamine, and methionine [13]]. Our study also found some new potential biomarkers, which greatly enriched the database of potential markers of sarcopenia and provided more directions for the diagnosis of sarcopenia. At the same time, we found that some potential biomarkers we detected this time were also found in previous experiments, which further shows that metabolomics has a certain repeatability in the study of sarcopenia. It also shows that these repeatedly verified potential biomarkers have great potential to become markers for the diagnosis of sarcopenia.

We showed that lipids are the most crucial group of altered metabolites in sarcopenia subjects compared to the control group. As recent study showed, lipids are an important category of metabolites and may play a significant role in the development and progression of age-related sarcopenia [13]. In muscle, lipids are stored inside muscle fibers (intramuscular lipids), outside muscle fibers (perimuscular and intermuscular lipids), or in the plasma membrane [20, 21]. In addition, intramuscular as well as perimuscular and intermuscular lipids can also be associated with the pathological condition of sarcopenia [21].

This targeted metabolomics study shows that low plasma lysophosphatidylcholine (LysoPC) was significantly correlated with sarcopenia, and LysoPC(17:0) might be one of the most representative potential plasma biomarkers of sarcopenia. PCs undergo conversion to LysoPC through the action of phospholipase A2 [21]. Consequently, the observed reduction in LysoPC levels may directly stem from the decrease in PC levels. PC, predominantly found in mitochondrial membranes, plays a crucial role in maintaining mitochondrial function [22]. Therefore, dysregulation of PC, leading to mitochondrial dysfunction, is likely to contribute to the onset and progression of various diseases [23]. Our findings are consistent with previous research in which LysoPC was associated with sarcopenia and its components in older men [9, 10]. LysoPC, a major class of glycerophospholipids in human plasma, are implicated in insulin resistance or inflammation [24]. As noted previously, skeletal muscle insulin resistance and inflammation were implicated in the pathogenesis of sarcopenia [25]. In addition, another study observed that in mice, diet-induced reductions in LysoPC may promote the loss of skeletal muscle force-generating capacity [26]. The findings highlight a remarkable correlation between LysoPC and maximal force. The LysoPC-mediated control of fiber cross-sectional area may potentially affect muscle protein turnover. Future studies will pursue the mechanism between LysoPC and sarcopenia.

In the present study, we found that L-2-amino-3-oxobutanoic acid may be a potential research focus in the pathogenesis of sarcopenia. However, a direct observation of L-2-amino-3-oxobutanoic acid in sarcopenia has never been reported. To the best of our knowledge, only two studies have shown related results about L-2-amino-3-oxobutanoic acid, which was associated with diabetes [27] and liver injury [28]. In the glycine, serine, and threonine metabolism pathway, L-2-amino-3-oxobutanoic acid is the downstream metabolite of glycine [28]. Glycine has previously been shown to reduce plasma insulin and fat mass in rodents [29]. Collectively, glycine’s impact on adiposity and insulin resistance can potentially be explained by improved insulin sensitivity and/or increased antioxidative and anti-inflammatory capacity [30]. Further metabolomics studies are needed to investigate the mechanisms involved in these processes.

Palmitic acid is the free saturated fatty acid with the highest level in blood. Although often considered to have adverse effects on chronic diseases in adults, palmitic acid is an essential component of cell membranes and secretory and transport lipids, with crucial roles in protein palmitoylation and palmitoylated signaling molecules [31]. There are extremely limited data on the relative effects of palmitic acid on sarcopenia. In the present study, it was found that the plasma levels of palmitic acid were decreased in the sarcopenia group, which was consistent with a previous study that conducted lipidomics analysis in sarcopenia and mild cognitive impairment patients [32] and based on GC–MS methods in stroke patients [33]. The palmitic acid tissue content seems to be controlled around a well-defined concentration. Particular physiopathological conditions and nutritional factors may result in an increased in the tissue content of palmitic acid and disrupted homeostatic control of its tissue concentration. Studies have shown that palmitic acid can activate NF-κB and promote massive release of inflammatory cytokines [34]. Inflammation is one of the important pathological factors of sarcopenia. Further research is needed to explore whether lower levels of palmitic acid are associated with related pathways of inflammation and oxidative stress, causing sarcopenia.

In our study, we observed that seven PC species [(14:0/20:2(11Z,14Z)), (16:0/20:3(8Z,11Z,14Z)), (18:2(9Z,12Z)/0:0), (P-18:0/16:0), (18:1(9Z)/P-18:1(9Z)), (17:2(9Z,12Z)/0:0), and (P-18:0/20:4(5Z,8Z,11Z,14Z))] were significantly decreased in sarcopenia subjects compared to control subjects. However, a previous study examining the relationship between resting phosphorus metabolites and skeletal muscle mass observed that older adults with sarcopenia had elevated levels of PCs [35]. The reasons for the variation in PCs may be attributed to the number of unsaturated double bonds. As Wang et al. reported, a decrease in PCs with more unsaturated double bonds might have a close relationship with an elevated risk of sarcopenia in elderly populations [32]. Furthermore, evidence convinced us that PCs containing more double bonds decreased in FABP3-overexpressing muscles (FABP3 is recognized as a valuable target for sarcopenia), whereas PCs with fewer double bonds increased [36]. Low levels of PCs might contribute to the accumulation of enlarged mitochondria that might become damaged and resist normal degradation through the autophagosomal/lysosomal pathway to produce high amounts of reactive oxygen species contributing to the aging process. In addition, decreased PC levels in the endoplasmic reticulum could induce endoplasmic reticulum stress, and the endoplasmic reticulum stress response pathway would slow the rate of protein synthesis contributing to the sarcopenia process [37]. However, further evidence is required regarding this new speculation.

PI (20:4(8Z,11Z,14Z,17Z)/18:0) was downregulated in the sarcopenia group compared with the control group in the present study. Research is lacking regarding a direct relationship between PI (20:4(8Z,11Z,14Z,17Z)/18:0) levels and sarcopenia. PI may be closely associated with the development of sarcopenia. The PI3K/Akt signaling pathway, a key molecular signal transduction pathway composed of PI, is involved in the development of sarcopenia. Studies have confirmed that deactivated PI3K/Akt during atrophy may not only lead to decreased protein synthesis but may also cause increases in the protein degradation rate, actin cleavage, and expression of muscle ubiquitin ligases in cell culture models of atrophy [38]. As a type of sphingolipid, sphingomyelin in mammalian cells is colocalized with cholesterol mainly in the plasma membrane and in lysosomal and Golgi membranes. Our findings are consistent with previous research in which sphingolipids were downregulated in the sarcopenia groups compared with controls [32]. Moreover, a basic research has demonstrated that enhanced absorption of sphingomyelin could increase muscle mass both in vitro and in vivo, thus protecting against or treating of sarcopenia [39].

Interestingly, we found that the level of ethylamine in the sarcopenia group was relatively high. There have been no studies of the direct effects of ethylamine on sarcopenia. The blood ethylamine levels were a possible indicator of the presence of L-theanine, which is one of the bioactive amino acids contained in green tea [40]. Chen et al. indicated that dietary L-theanine supplementation promoted skeletal muscle fiber transition from type II to type I [41]. Sarcopenia was characterized by a significantly lower type II fiber diameter [42]. However, another study investigated the association between serum ethylamine levels and the development of type 2 diabetes and showed that L-theanine may improve insulin resistance by modifying the inflammatory response [40]. The reduced insulin-mediated suppression of proteolysis is associated with sarcopenia [43]. Therefore, the exact mechanism needs further investigation. Further studies will be needed to corroborate these findings in other populations. Finally, the plasma levels of palmitamide in sarcopenia patients were decreased, but due to the lack of literature reports, the relationship between palmitamide and sarcopenia still needs to be investigated.

Despite our efforts, some limitations exist. First, due to the cross-sectional design, we were unable to predict sarcopenia prognosis or determine the causal relationship between plasma differential metabolites and sarcopenia. Second, given our relatively small sample size, we acquired normal control subjects matched solely for age and sex, without considering BMI. Statistical significance was determined using uncorrected *p*-values, potentially constraining our ability to conclusively identify metabolites associated with sarcopenia. Subsequent research with larger samples should address this more rigorously, incorporating additional confounding factors (including BMI) for propensity score calculation and employing multiple comparison correction.

## Conclusion

The metabolic analysis of sarcopenia is the first study by utilizing untargeted GC/LC–MS of plasma to obtain more comprehensive metabolomics characteristics and to screen out a large number of potential biomarkers with significant differences. Through multivariate data analysis, most of these metabolic markers are related to disorders of lipid metabolism and amino acid metabolism in sarcopenia. Fifty-five metabolites by GC–MS and 85 metabolites by LC–MS were judged to have great potential as biomarkers of sarcopenia. Among them, LysoPC(17:0), L-2-amino-3-oxobutanoic acid, and palmitic acid specifically emerged as potential predictors for the development of sarcopenia. The biological mechanisms underlying the relationship between these metabolites and sarcopenia traits still need to be clarified in future studies.

## Data Availability

The data underlying this article will be shared at reasonable request to the corresponding author.

## Abbreviations

Akt: α-Serine/threonine-protein kinase
ASM: Appendicular skeletal muscle mass
AUC: Area under the curve
AWGS: Asian working group for sarcopenia
BIA: Bioelectrical impedance analysis
BMI: Body mass index
CI: Credibility interval
ESI: Electrospray ionization
FC: Fold change
GC: Gas chromatography
GDS: Geriatric depression scale
Ht: Height
IPAQ: International physical activity questionnaire
LC: Liquid chromatography
LysoPCs: Lysophosphatidylcholines
MNA: Mini nutritional assessment-short form
MS: Mass spectrometry
NC: Normal control
OPLS-DA: Orthogonal partial least squares–discriminant analysis
PC: Phosphatidylcholine
PI: Phosphatidylinositol
PI3K: Phosphatidylinositide 3-kinases
ROC: Receiver operating characteristic
RSD: Relative standard deviation
RT: Retention time
SM: Sphingomyelin
VIP: Variable importance in projection
